# Cell Line Derived Multi-Gene Predictor of Pathologic Response to Neoadjuvant Chemotherapy in Breast Cancer: A Validation Study on US Oncology 02-103 Clinical Trial

**DOI:** 10.1186/1755-8794-5-51

**Published:** 2012-11-16

**Authors:** Kui Shen, Yuan Qi, Nan Song, Chunqiao Tian, Shara D Rice, Michael J Gabrin, Stacey L Brower, William Fraser Symmans, Joyce A O’Shaughnessy, Frankie A Holmes, Lina Asmar, Lajos Pusztai

**Affiliations:** 1 Department of Product Development, Precision Therapeutics, Inc, 2516 Jane Street, Pittsburgh, PA 15203, USA; 2 Department of Bioinformatics and Computational Biology, University of Texas MD Anderson Cancer Center, Houston, TX 77230, USA; 3 Department of Pathology, University of Texas MD Anderson Cancer Center, 1515 Holcombe Blvd. Unit 0085, Houston, TX 77030, USA; 4 Baylor Sammons Cancer Center, Texas Oncology, US Oncology, Baylor-Sammons Cancer Center, 3535 Worth St., Collins 5, Dallas, TX 75246, USA; 5 Texas Oncology, US Oncology, 925 Gessner St #550, Houston, TX 77024, USA; 6 Biostatistics and Medical Writing, US Oncology Research, US Oncology, The Woodlands, Woodlands, TX 77380, USA; 7 Department of Breast Medical Oncology, Unit 1354, The University of Texas M. D. Anderson Cancer Center, PO Box 301439, Houston, TX 77230, USA

**Keywords:** Multi-gene predictor of chemosensitivity, Breast cancer, Cell lines

## Abstract

**Background:**

The purpose of this study is to assess the predictive accuracy of a multi-gene predictor of response to docetaxel, 5-fluorouracil, epirubicin and cyclophosphamide combination chemotherapy on gene expression data from patients who received these drugs as neoadjuvant treatment.

**Methods:**

Tumor samples were obtained from patients with stage II-III breast cancer before starting neoadjuvant chemotherapy with four cycles of 5-fluorouracil/epirubicin/cyclophosphamide (FEC) followed by four cycles of docetaxel/capecitabine (TX) on US Oncology clinical trial 02-103. Most patients with HER-2-positive cancer also received trastuzumab (H). The chemotherapy predictor (TFEC-MGP) was developed from publicly available gene expression data of 42 breast cancer cell-lines with corresponding in vitro chemotherapy sensitivity results for the four chemotherapy drugs. No predictor was developed for treatment with trastuzumab. The predictive performance of TFEC-MGP in distinguishing cases with pathologic complete response from those with residual disease was evaluated for the FEC/TX and FEC/TX plus H group separately. The area under the receiver-operating characteristic curve (AU-ROC) was used as the metric of predictive performance. Genomic predictions were performed blinded to clinical outcome.

**Results:**

The AU-ROC was 0.70 (95% CI: 0.57-0.82) for the FEC/TX group (n=66) and 0.43 (95% CI: 0.20-0.66) for the FEC/TX plus H group (n=25). Among the patients treated with FEC/TX, the AU-ROC was 0.69 (95% CI: 0.52-0.86) for estrogen receptor (ER)-negative (n=28) and it was 0.59 (95% CI: 0.36-0.82) for ER-positive cancers (n=37). ER status was not reported for one patient.

**Conclusions:**

Our results indicate that the cell line derived 291-probeset genomic predictor of response to FEC/TX combination chemotherapy shows good performance in a blinded validation study, particularly in ER-negative patients.

## Background

Neoadjuvant (pre-operative) chemotherapy is increasingly used in the treatment of early stage breast cancer; however, the extent of clinical and pathologic response varies widely among patients. Identifying individuals that are most responsive to standard chemotherapy regimens would be clinically useful since it could maximize the benefit among responders and minimize unnecessary side effects from ineffective therapy for those who are predicted to be less sensitive. The latter could be encouraged to participate in clinical trials with newer regimens.

Gene expression profiling has shown that breast cancer is a heterogeneous disease at the molecular level [1,2], which underlies its variable responses to therapy. Several investigators have attempted to develop multi-gene predictors (MGP) of response by correlating baseline, pretreatment tumor gene expression profiles with observed clinical responses to specific treatments [3-5]. While attractive, this approach has its limitations. Discovery sample size is usually small due to the necessity to prospectively collect tissue for analysis, and treatment sensitivity is not distributed equally among various phenotypic subtypes of breast cancer.

Several investigators suggested that MGPs less biased by phenotype may be developed from cell line models. This approach relies on selecting probe sets that are associated with response from cell lines exposed to drugs in vitro and using these informative probe sets to train an MGP model on the cell line data. Several different cell line-derived drug response predictors were developed using the NCI-60 cell lines (http://dtp.nci.nih.gov/docs/cancer/cancer_data.html); however, when these predictors were applied to human data to predict response, the results were mixed [6,7]. Generally, it is believed that predictors developed from cell lines that represent a single histological type of cancer may perform better when applied to the corresponding human cancer type than predictors derived from cell lines of mixed origin [8]. Validation results in human data remain controversial. For example, an MGP derived from melanoma cell lines was shown to successfully predict response to temozolomide in melanoma patients [9], whereas an MGP derived from breast cancer cell lines was unable to predict clinical outcomes for breast cancer patients treated by paclitaxel followed by 5-FU, doxorubicin, and cyclophosphamide [10]. Therefore, it is of interest to continue investigating the feasibility of developing MGPs using cell lines of the same tumor type.

In this study, we developed an MGP from a panel of breast cancer cell lines treated with the 4-drug combination in vitro and validated its predictive ability on patient samples obtained during a prospective neoadjuvant clinical trial. We tested 42 breast cancer cell lines for their sensitivity to the combination of docetaxel, epirubicin, 5-fluorouracil and cyclophosphamide using an in vitro cell viability assay and used publicly available gene expression data that match these cell lines to develop a multi-gene predictor. The predictive performance of this MGP was validated independently by MD Anderson Cancer Center on patients from the US Oncology 02-103 clinical trial. Clinical results of US Oncology 02-103 have been previously presented [11,12].

## Methods

### Patients and Samples

US Oncology 02-103 was a single arm neoadjuvant trial involving women with stage II/III breast cancer. Patients with Human Epidermal Growth Factor Receptor 2 (HER2)-negative cancer received FEC 100, which consisted of 5-fluorouracil (Adrucil [5-FU], 500 mg/m^2^) + epirubicin (Ellence, 100 mg/m^2^) + cyclophosphamide (Cytoxan, 500 mg/m^2^) IV on Day 1 every 21 days (x 4 cycles, 12 weeks total) followed by wTX, Taxotere (35 mg/m^2^) weekly Days 1 and 8 every 21 days (x 4 cycles, 12 weeks total), (FEX/TX group). Patients with HER2-positive tumors received FEC 75, which consisted of 5-fluorouracil (Adrucil [5-FU], 500 mg/m^2^) + epirubicin (Ellence, 75 mg/m^2^) + cyclophosphamide (Cytoxan, 500 mg/m^2^) IV on Day 1 every 21 days with trastuzumab (Herceptin) 4 mg/kg IVx1 as initial loading dose on Day 1 followed by 2 mg/kg IV weekly x12 (x4 cycles, 12 weeks total), followed by wTX, Taxotere (35 mg/m^2^) weekly Days 1 and 8 every 21 days (x 4 cycles, 12 weeks total) and Herceptin 2 mg/kg weekly x12, (FEC/TX plus H group). HER2 status was assessed by immunohistochemistry (IHC) or fluorescent in situ hybridization (FISH). IHC ≥3+ was considered positive and IHC 1+ or 2+ was confirmed by FISH. The primary study endpoint was pathologic complete response (pCR) rate defined as no viable invasive cancer in the breast and lymph nodes after completion of neoadjuvant chemotherapy. The US Oncology 02-103 clinical trial was approved by the institutional review board of the practice group and all patients provided written informed consent to participate in the therapeutic trial and to provide a specimen for genomic analysis of the cancer. Pre-treatment fine-needle aspiration (FNA) specimens were obtained and immediately placed in RNAlater (Ambion, Austin, TX), and shipped to the University of Texas MD Anderson Cancer Center (UTMDACC) for RNA extraction and gene expression profiling with Affymetrix HU133A gene chips (Affymetrix, Santa Clara, CA) as described previously [4]. Tissue analysis was approved by the Institutional Review Board of UT MDACC. Full gene expression 132 data is available at Gene Expression Omnibus under accession number GSE42822.

### In vitro chemosensitivity testing of breast cancer cell lines

Forty-two breast cancer cell lines were obtained from ATCC (Manassas, VA) or DSMZ (Braunschweig, Germany). Cell lines were selected primarily based on the availability of publicly available expression data, availability for commercial purchase, and the compatibility with use in an in vitro chemosensitivity assay. All cell lines were maintained in RPMI 1640 (Mediatech, Herndon, VA) containing 10% FBS (HyClone, Logan, UT) at 37°C in 5% CO_2_. Upon reaching approximately 80% confluence, each cell line was trypsinized and seeded into 384-well microtiter plates (Corning, Lowell, MA) for in vitro chemotherapy sensitivity testing.

Cell lines were treated with the combination of docetaxel (0.1nM – 25nM), 5-fluorouracil (0.1 μM - 50 μM), epirubicin (0.7nM – 13.5 μM) and preactivated cyclophosphamide (4-hydroperoxycyclophosphamide) (0.2 μM – 13.6 μM) (TFEC) to simulate the treatment protocol of FEC followed by TX in the US Oncology 02-103 clinical trial [13]. Ten serial dilutions prepared in 10% RPMI 1640 media, along with a media control that did not contain drugs, were added in triplicate to each cell line. The combination treatment was composed of equal volumes of each drug at each dose number, i.e. combination treatment dose 1 contained equal volumes of the dose 1 concentration of each component drug, combination treatment dose 2 contained equal volumes of the dose 2 concentrations of each component drug, and so on. The clinical formulation of docetaxel was used, which was supplied as a concentrated solution of docetaxel in polysorbate 80 plus a vial of diluent (13% w/w ethanol in water for injection). Cells were incubated for 72 hours at 37°C in 5% CO_2_. Non-adherent cells and medium were then removed from each well. The remaining adherent cells were fixed in 95% ethanol and stained with DAPI (Molecular Probes, Eugene, OR). A proprietary automated microscope was used to count the number of stained cells remaining after drug treatment [14]. A survival fraction (SF) at dose *i* (*i* = 1, 2, …, 10) was calculated as $SF_{i}=\frac{mean_{\text{drug}}^{\left(i\right)}}{mean_{\text{control}}}$, where *mean*^*(i)*^_*drug*_ is the average of the number of surviving cells in the drug treated wells at dose *i*, and *mean*_*control*_ is the average number of living cells in the control wells. The area under the dose–response curve, which is the summation of SF values over 10 doses, *AUC=ΣSF*_*i*_, was used to quantify the sensitivity of each cell line to the treatment of TFEC, with lower AUC score indicating greater sensitivity.

### Statistical Analysis

#### Development of the TFEC-MGP

Gene expression profiles for the 42 breast cancer cell lines [15] generated with Affymetrix HG-U133 Plus 2.0 Array (Affymetrix, Santa Clara, CA), were downloaded from the Gene Expression Omnibus database (Accession number GSE12777). The RMAExpress V1.05 software package (http://rmaexpress.bmbolstad.com) [16] was used to generate probe level intensities by setting the operating parameters as: Background adjust: Yes; Normalization: Quantile; Summarization method: Probe level model. The probe level intensities were log_2_-transformed before further analyses. Non-specific filtering was applied to remove probe sets having small variation (interquartile range < 0.5) or low expression values (median < log_2_(100)) across all cell lines. The expression values were then standardized to mean zero and standard deviation one for each cell line.

The MGP was developed based on supervised principal components regression [17,18] and implemented by using Superpc V1.05 software package (http://www-stat.stanford.edu/~tibs/superpc) under the programming environment R 2.11.1 (http://www.r-project.org). Code is provided in the Additional file 1. Briefly, univariate linear regression analysis was first conducted to calculate the association between the cell lines’ AUC scores derived from the dose response curves and the expression values for each probe set. Probe sets with a regression coefficient larger than the threshold (1.8) estimated by 10-fold cross-validation were selected and their expression values were used for principle component analysis. The first principal component was then chosen as an independent variable in a linear regression model to predict the patient’s chemotherapy response. A lower prediction score corresponds to a greater chemotherapy sensitivity and therefore higher likelihood of achieving pCR. CEL files from cancer biopsies were provided to PTI by UTMDACC without any accompanying clinical information. These array data were processed by RMA using the same procedure as the one used for cell lines. Prediction scores were calculated by investigators at PTI and returned to collaborators at UTMDACC to calculate AU-ROC curves and compare scores between patients with pCR versus residual disease (RD) response outcome.

To understand the functions of these probe sets, gene set enrichment analysis was performed based on the c2 collection of molecular signatures database v3.0 provided by Broad Institute (http://www.broadinstitute.org/gsea/msigdb/index.jsp). The q-value of each gene set was calculated by the permutation test. Gene sets with q-value less than 0.1 were considered to be enriched.

### Clinical validation of MGP

MGP scores were compared between patients with pCR and RD using the non-parametric Wilcoxon test. The scores were used as a continuous variable to perform receiver-operator characteristics curve (ROC) analysis to evaluate the predictive performance of the MGP. Univariate and multivariate logistic regression analyses were also performed including ER status, nodal status and tumor grade as categorical variables and age, tumor size and the MGP score as continuous variables. To control for the confounding effect of trastuzumab, analyses were done separately for patients treated with FEC/TX plus H and patients treated with FEC/TX.

## Results

### Gene signature for TFEC-MGP

In vitro chemosensitivity results, represented by the area under the dose–response curve values to TFEC treatment, were determined for each of the 42 breast cancer cell lines (Additional file 2. Table S1). Through univariate analysis, 633 genes were identified to be significantly correlated with drug response (q < 0.01). Among them, the top 291 probe sets with a regression coefficient larger than the threshold estimated by 10-fold cross-validation were included in the final model. The corresponding gene symbols and functions of these 291 probe sets are listed in the Additional file 2: Table S3. Functional analysis indicated that these genes are involved in different biological process, including cell cycle, transcription, translation, immune response, and metabolism (Additional file 2: Table S4). Noticeably, among the identified 44 datasets, several of them are related to proto-oncogene MYC and BRCA1, which are very well studied genes that play important roles in breast cancer pathogenesis.

### Clinical validation of TFEC-MGP

A total of 184 pre-treatment specimens were obtained during the trial. Sufficient and high-quality RNA were isolated from 111 specimens. Of these, 91 specimens were included in the final analysis. Reasons for exclusion included failure of cRNA generation (n=8), failure to meet quality control standards for array analysis (n=8), and lack of outcome data (n=4). The unexpectedly low success rate for RNA isolation may be due to various reasons, including use of a technology-intense procedure in community-based offices and, as a result, a strong learning curve effect on the RNA yields and success rate. The patient demographic characteristics of this study population were similar to those from the trial as a whole with respect age, tumor size, node status and ER status, but not to histologic grade and HER-2 status (Additional file 2: Table S2). The difference is likely due to a relatively large number of patients with unknown tumor grade and HER-2 information. Of the 91 patients eligible for our study, 66 received treatment with FEC/TX and 25 received treatment with FEC/TX plus H. Table 1 details the demographic characteristics for these patients.

**Table 1:** Clinical and demographic characteristics of breast cancer patients treated with FEC/TX (n=66) and FEC/TX plus H (n=25)

**Characteristic**	**66 patients treated with FEC/TX**		**25 patients treated with FEC/TX plus H**	
	**pCR No. cases (%)**	**RD No. cases (%)**	**pCR No. cases (%)**	**RD No. cases (%)**
All patients	25 (100)	41 (100)	12(100)	13 (100)
Age in yrs				
Median (range)	49 (34-69)	49 (26- 67)	50 (37-59)	49 (39-64)
Histology				
Invasive ductal (IDC)	24 (96.0)	38 (92.7)	12 (100.0)	12 (92.3)
Invasive lobular (ILC)	0 (0.0)	1 (2.4)	0 (0.0)	0 (0.0)
Invasive mucinous (IMC)	0(0.0)	1 (2.4)	0(0.0)	0 (0.0)
NOS	1 (4.0)	1 (2.4)	0 (0.0)	1 (7.7)
Clinical tumor size at baseline				
T1	0 (0)	1 (2.4)	0 (0.0)	0 (0.0)
T2	9 (36.0)	14 (34.1)	4 (33.3)	7 (53.8)
T3	14 (56.0)	23 (56.1)	6 (50.0)	4 (30.8)
T4	2 (8.0)	3 (7.3)	2 (16.7)	2(15.4)
Clinical node status at baseline				
N0	8 (32.0)	15 (36.6)	2 (16.7)	4 (30.8)
N1	12 (48.0)	22 (53.7)	7(58.3)	4 (30.8)
N2	2 (8.0)	2 (4.9)	1 (8.3)	4 (30.8)
N3	3 (12.0)	1 (2.4)	1 (8.3)	0 (0.0)
Unkown			1 (8.3)	1 (7.7)
Histologic Grade				
1 & 2	4 (16.0)	10 (24.4)	6 (50.0)	3 (23.1)
3	14 (56.0)	26 (63.4)	4 (33.3)	9 (69.2)
Unknown	7 (28.0)	5 (12.2)	2 (16.7)	1 (7.7)
ER status				
Positive^1^	8 (32.0)	20 (48.8)	2 (1.7)	8 (61.5)
Negative	17 (68.0)	20 (48.8)	10 (76.9)	5 (38.5)
Unknown	0 (0.0)	1 (2.4)		
HER-2 status				
Positive ^2^	5 (20.0)	5 (12.2)	13 (100)	12 (92.3)
Negative	20 (80.0)	33 (80.5)	0 (0.0)	1 (7.7)
Unknown	0 (0.0)	3 (7.3)	0 (0.0)	0 (0.0)

^1^Cases where >10% of tumor cells stained positive for ER with immunohistochemistry were considered positive. ^2^Cases that showed either 3+ IHC staining or had gene copy number >2.0 were considered HER2-positive.

**Figure 1:**
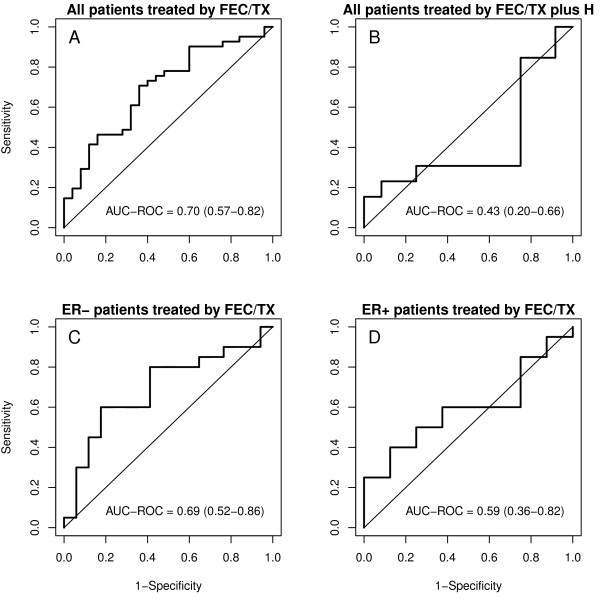
**Prediction results for patients receiving FEC/TX with and without H treatment. A**: ROC curve for TFEC-MGP for all patients who did not receive H treatment. **B**: ROC for TFEC-MGP for all patients who received H treatment. **C**: ROC curve for TFEC-MGP for ER-negative patients who did not receive H treatment. **D**: ROC curve for TFEC-MGP for ER-positive patients who did not receive H treatment.

Among the FEC/TX-treated cases (n = 66), the mean MGP scores were significantly lower in pCR (0.23, 95% CI: 0.19-0.26) compared to RD (0.29, 95% CI: 0.26-0.32) cohorts (Wilcoxon rank test: p<0.01). The AU-ROC was 0.70 (95% CI: 0.57-0.82) across all patients including both ER-positive and ER-negative cancers (Figure 1). In contrast, among the HER2-positive FEC/TX plus trastuzumab treated cases, the mean MGP scores for cases with pCR and RD were identical, 0.29 and 0.28, respectively (p=0.57) and the AU-ROC was 0.43 (95% CI: 0.20-0.66).

We also examined predictive performance separately for the ER-positive and ER-negative cancers that were treated with FEC/TX. The AU-ROC was 0.59 (95% CI: 0.36-0.82) for the ER-positive subgroup (n = 37) and 0.69 (95% CI: 0.52-0.86) for the ER-negative subgroup (n = 28) (Figure 1) (note: ER status was not reported for one patient). This finding suggests that this particular MGP may have a discriminating value in ER-negative and HER-2 negative tumors.

Logistic regression analysis was performed to further assess the performance of the TFEC-MGP in predicting pCR. Univariate analysis revealed that only the MGP score was significantly associated with pCR (p<0.0001; Table 2). Regression analysis for the FEC/TX plus H group found no significant association between the TFEC-MGP prediction scores and pCR status (Table 3).

**Table 2:** Univariate and multivariate analysis results for the FEC/TX group (n=66)

**Factors**	**Univariate logistic regression analysis**		**Multivariate logistic regression analysis**	
	**OR (95% CI)**	**p-value**	**OR (95% CI)**	**p-value**
Age	1 .00 (0.95, 1.05)	0.94	1.01 (0.94, 1.08)	0.8
ER	0.47 (0.17, 1.34)	0.16	1.16 (0.19,7.18)	0.87
PR	0.51 (0.18, 1.42)	0.20	0.87 (0.14, 5.48)	0.88
Nodal	1.27 (0.44, 3.67)	0.65	1.92 (0.46, 8.01)	0.37
T	1.10 (0.50, 2.43)	0.82	1.3 (0.49, 3.45)	0.60
Grade	1.35 (0.36, 5.09)	0.66	1.07 (0.24, 4.77)	0.92
MGP scores	0.00 (0.00, 0.2)	0.01	0.00 (0.00, 7.28)	0.15

OR: odds ratio for having pCR vs. RD; CI, confidence interval; p-value. The comparison vs. reference state for the categorical variables were ER, estrogen receptor (positive vs. negative) PR, progesterone receptor (positive vs. negative); Nodal (positive vs. negative); T (Tumor size); Grade (grade 3 vs. 2) and MGP scores.

**Table 3:** Univariate and multivariate analysis results for FEC/TX plus H group (n=25)

	**Univariate logistic regression analysis**		**Multivariate logistic regression analysis**	
	**OR (95% CI)**	**p-value**	**OR (95% CI)**	**p-value**
Age	0.99 (0.88, 1.10)	0.83	0. 91 (0.68, 1.22)	0.53
ER	0.13 (0.02, 0.82)	0.03	0.01 (0.00, 2.21)	0.09
Nodal	2.25 (0.32, 15.76)	0.41	0.15 (0.00, 7.94)	0.26
T	1.53 (0.5, 4.63)	0.45	0.66(0.07, 6.68)	0.73
Grade	0.22 (0.04, 1.37)	0.11	0.05 (0 .00, 2.33)	0.13
MGP scores	11.93 (0.00, 1.27E+06)	0.67	1.85E+06 (0.00, 1.35E+16)	0.21

OR: odds ratio for having pCR vs. RD; CI, confidence interval; p-value. The comparison vs. reference state for the categorical variables were ER, estrogen receptor (positive vs. negative) Nodal (positive vs. negative); T (Tumor size); Grade (grade 3 vs. 2) and MGP scores.

## Discussion

In this study, we developed a TFEC-MGP from breast cancer cell lines by associating in vitro drug response data with gene expression profiling data. Independent, blinded validation of this MGP using clinical data from US Oncology 02-103 clinical trial indicated that this cell line derived MGP was able to differentiate between patients who would experience pCR and those who would have RD after neoadjuvant treatment with FEC followed by TX. This result demonstrated the feasibility of developing an MGP predicting pCR of breast cancer patients using chemoresponse data and gene expression profiling from breast cancer cell lines.

These results differ from a previous study that was not successful in developing an MGP from breast cancer cell lines in several important features [10]. This study used a larger number of cell lines for discovery (42 versus 19). In addition, in this study, cells were exposed to a drug combination (instead of single agents) and in vitro chemosensitivity was assessed through direct measurements of cell death (as opposed to biochemical assays). Different statistical methods were also used to generate our prediction model. An important strength of this study is that prediction results were generated blinded to any outcome information.

Clinical variables such as ER, PR, HER2 and tumor grade are well known to be associated with chemotherapy responses in breast cancer but these were not significant in univariate analysis in this study. It is desirable to develop MGPs that provide independent information of these clinical variables. Ideally, an MGP would be developed for each molecular subset of breast cancer. Although sufficient numbers of suitable cell lines for each tumor subtype are not yet established to allow MGP discovery by subtype, our study indicates that informative data could still be gleaned from combined analysis of all different breast cancer cell lines. Of note, the subset analysis stratified by ER status revealed that this MGP may provide information independent of ER status, indicating that the MGP may have predictive value in both ER-positive and ER-negative patients. This finding is particularly of interest for ER-negative patients, whose clinical outcomes are difficult to predict.

It is also notable that the MGP developed for the FEC/TX treatment arm did not have prediction benefit for patients in the FEC/TX plus trastuzumab treatment arm. This may be due to the small number of patients in the FEC/TX plus H group, making it highly unlikely to find an effect due to lack of power. Moreover, while trastuzumab can substantially improve the chemotherapy response for HER2-positive patients [19], the MGP developed in the present study did not include drug response data for trastuzumab; this may also lead to the poor performance in FEC/TX plus H treatment arm.

To understand how the predictive performance of an MGP developed from cell lines compares with the performance of other signatures developed from patients, we compared our 291-gene signature with three well-recognized genomic signatures which were developed from patients: 70-gene signature [20], ROR (Risk of Relapse) score which was only based on intrinsic subtype (ROR-S) [21], and ROR score which combines information from subtype and proliferation genes (ROR-P) [21]. The prediction results based on these 4 genomic signatures are highly correlated to each other (data not shown). Moreover, their performance of predicting pathological complete response (pCR) is very similar. For all patients treated by FEC/TX (n = 69), the AUC-ROC for the 291-gene signature, 70-gene signature, ROR-S, and ROR-P are 0.70 (0.57 – 0.82), 0.68 (0.81-0.55), 0.77 (0.65-0.89), and 0.74 (0.64-0.89), respectively. This observation indicates that a genomic predictor developed from cell lines performs similarly to other genomic predictors derived from patients, which should be considered along with the other advantages of cell line-based MGP development.

This study also has several limitations, foremost the validation sample size was small and therefore confidence intervals around the AU-ROC estimates were broad. Secondly, we recognize the differences in chemotherapy regimen between our in vitro assay and the clinical treatment that patients received. For example, the serial dilution of TFEC in vitro is not an attempt to simulate the FEC/TX regimen that was used clinically in USO 02-103. In addition the concurrent administration of docetaxel in vitro may not be equivalent to the subsequent administration of docetaxel in vivo. This may affect the performance of the developed MGP. Thirdly, in our analysis, patient response is divided into either pathological complete response (pCR) or residual disease (RD). However, most cases with RD have some degree of tumor response. An analysis based on RD score (tumor residual evaluated as a continuous variable) would be ideal; unfortunately, this information was not collected in our dataset. Finally, we did not establish MGP cut off values in this study to define responder versus non-responder categories for patients, which would require a substantially larger sample size and a separate independent cohort to test the validity of the selected threshold. In the absence of large validation cohorts it remains unknown whether the true predictive performance of this assay is sufficiently high or not for clinical use.

## Conclusions

In summary, this study provides a proof of principle that an MGP for TFEC treatment can be generated from breast cancer cell lines with known response to this combination chemotherapy which remains informative when applied to human patient data. It is particularly encouraging that the predictor has the trend of performing better for ER-negative tumors, a subset of breast cancers for which the development of pharmacogenomic response predictors has proven to be difficult in the past [22].

## Competing interests

KS, NS, CT, SDR, MJG, and SLB are employees of Precision Therapeutics Inc. WFS has ownership interest (including patients) and is consultant/Advisory board of Nuvera Biosciences. JAO is consultant of Sanofi-aventis and Roche. YQ, FAH, LA, and LP have no conflict.

## Authors’ contributions

SDR performed drug response assay to measure cell line drug response. JAO, FAH, WFS, LA, LP gathered samples and clinical information. KS, YQ, NS analyzed the data. KS, NS, CT, MJG, SLB, LP drafted and revised manuscript. All authors read and approved the final manuscript.

## Pre-publication history

The pre-publication history for this paper can be accessed here:

http://www.biomedcentral.com/1755-8794/5/51/prepub

## Supplementary Material

Additional file 1 Code of developing TFEC-MGP from breast cancer cell lines. Click here for file

Additional file 2 **Table S1.** AUC values for 42 breast cancer cell lines treated by TFEC. **Table S2.** Clinical and demographic characteristics of breast cancer patients in this study compared with patients in USO trial. **Table S3.** Two hundred and ninety one affymetrix probes used in TFEC-MGP. The gene symbols and descriptions of these probes were obtained by using broad institute’s molecular signatures database v3.0 (http://www.broadinstitute.org/gsea/msigdb/index.jsp). **Table S4.** Identified enriched pathways. Click here for file
