# Supplementary material for: Cell Line Derived Multi-Gene Predictor of Pathologic Response to Neoadjuvant Chemotherapy in Breast Cancer: A Validation Study on US Oncology 02-103 Clinical Trial
Source: BMC Med Genomics. 2012 Nov 16;5:51. doi: 10.1186/1755-8794-5-51 (PMC3536618; doi:10.1186/1755-8794-5-51)
Supplement: Additional file 2 — Table S1. AUC values for 42 breast cancer cell lines treated by TFEC. Table S2. Clinical and demographic characteristics of breast cancer patients in this study compared with patients in USO trial. Table S3. Two hundred and ninety one affymetrix probes used in TFEC-MGP. The gene symbols and descriptions of these probes were obtained by using broad institute’s molecular signatures database v3.0 (http://www.broadinstitute.org/gsea/msigdb/index.jsp). Table S4. Identified enriched pathways. [file 1755-8794-5-51-S2.docx]

**Supplemental Materials**

**Supplemental A: The algorithm for pathway enrichment analysis**

The microarray data was pre-processed including non-specific filtering as described in the manuscript.

1. Calculate $p(t_{g})$, the correlation coefficient of gene *g* by superPC, 1≤*g≤G*. Used the cutoff defined in the manuscript, the genes were divided into two groups, A: the genes whose correlation coefficient is larger than the cutoff (gene signatures for TFEC predictor) and B: the genes whose correlation coefficient is smaller than the cutoff.
2. Compute $P_{p}^{KS}$, the p-value of pathway p by testing the independence of the following 2x2 contingency Table using Fisher’s exact test. The c2 collection of molecular signatures database v3.0 provided by Broad Institute was chosen in this study. The gene sets whose size larger than 15 and less than 500 was used for further analysis.

|  | Genes in group A | Genes in group B | Total |
| --- | --- | --- | --- |
| In the pathway | *n_pd_* | *n_pd_^c^* | *n_p_* |
| Not in the pathway | *n_p_^c^_d_* | *n_p_^c^_d_^c^* | *n_p_^c^* |
| Total | *n_d_* | *n_d_^c^* | *N* |

1. Permute gene labels C times, and calculate the permuted statistics, $P_{p}^{KS(c)}$ *,* 1 *≤ c ≤ C.C* was set to 500 in this study.
2. Estimate the p-value of pathway *p* as $p(v_{p})=\sum_{c=1}^{C} \sum_{p'=1}^{P} I(P_{p'}^{KS(c)}\leq P_{p}^{KS})/C\cdot P$ and similarly calculate$v_{p}^{c}=\sum_{c'=1}^{C} \sum_{p'=1}^{P} I(P_{p'}^{KS(c')}\leq P_{p}^{KS(c)})/C\cdot P$.

5. Estimate $\pi_{0}$, the proportion of non-enriched pathways in the meta-analysis, as $\hat{\pi}_{0}=\frac{\sum_{p=1}^{P} I(p(v_{p})\in A)}{P\cdot l(A)}$. For simplicity, we set $\pi_{0}$ to 1 in this study.

6. Estimate q-value of pathway *p* as

$q(v_{p})=\hat{\pi}_{0}\sum_{c=1}^{C} \sum_{p'=1}^{P} I(P_{p'}^{KS(c)}\leq P_{p}^{KS})/C\cdot\sum_{p'=1}^{P} I(P_{p'}^{KS}\leq P_{p}^{KS})$.

**Supplementary Table 1. AUC values for 42 breast cancer cell lines treated by TFEC.**

|  | AUC for TFEC | ER | PR | HER2 |
| --- | --- | --- | --- | --- |
| AU565 | 3.627 | neg | neg | pos |
| BT20 | 4.799 | neg | neg | neg |
| BT474 | 6.730 | pos | pos | pos |
| BT483 | 6.285 | pos | pos | neg |
| BT549 | 4.154 | neg | neg | neg |
| CAL120 | 3.657 | neg | neg | neg |
| CAL51 | 2.796 | neg | neg | neg |
| CAL851 | 4.281 | neg | neg | neg |
| CAMA1 | 4.656 | pos | neg | neg |
| EFM19 | 6.992 | pos | pos | neg |
| EFM192A | 4.849 | pos | neg | pos |
| EVSAT | 3.411 | NA | pos | neg |
| HCC1143 | 5.068 | neg | neg | neg |
| HCC1395 | 3.853 | neg | neg | neg |
| HCC1419 | 6.826 | neg | neg | pos |
| HCC1428 | 6.707 | pos | pos | neg |
| HCC1500 | 7.266 | neg | neg | neg |
| HCC1569 | 5.014 | neg | neg | pos |
| HCC1806 | 2.726 | neg | neg | neg |
| HCC1937 | 4.490 | neg | neg | neg |
| HCC1954 | 3.516 | neg | neg | pos |
| HCC202 | 6.812 | neg | neg | pos |
| HCC38 | 3.822 | neg | neg | neg |
| HDQP1 | 4.106 | neg | neg | neg |
| JIMT1 | 4.113 | neg | neg | pos |
| KPL1 | 2.830 | pos | pos | pos |
| MCF7 | 4.761 | pos | pos | neg |
| MDAMB134VI | 5.056 | pos | neg | neg |
| MDAMB175VII | 7.942 | pos | neg | neg |
| MDAMB231 | 3.635 | neg | neg | neg |
| MDAMB361 | 7.735 | pos | neg | pos |
| MDAMB415 | 4.448 | pos | neg | neg |
| MDAMB436 | 4.678 | neg | neg | neg |
| MDAMB453 | 6.269 | neg | neg | neg |
| MDAMB468 | 3.178 | neg | neg | neg |
| MFM223 | 3.108 | neg | pos | neg |
| SKBR3 | 2.431 | neg | neg | pos |
| SW527 | 3.012 | NA | NA | NA |
| T47D | 3.792 | pos | pos | neg |
| UACC812 | 2.711 | pos | neg | pos |
| ZR751 | 6.975 | pos | neg | neg |
| ZR7530 | 6.429 | pos | neg | pos |

**Supplementary Table 2**. Clinical and demographic characteristics of breast cancer patients in this study compared with patients in USO trial.

|  | All USO  (n = 184) | MGP study  ( n = 91) |
| --- | --- | --- |
| Characteristic | No. cases (%) | No. cases (%) |
| **Age in yrs**  Median (range) | 50 (26-74) | 50(34-69) |
| **Histology** |  |  |
| Invasive ductal (IDC) | 164 (89.1) | 86 (94.5) |
| Invasive lobular (ILC) | 11 (6.0) | 1 (1.1) |
| Invasive mucinous (IMC) | 5 (2.7) | 1 (1.1) |
| NOS | 4 (2.2) | 3 (3.3) |
| **Clinical tumor size at baseline** |  |  |
| T1 | 5 (2.7) | 1 (1.1) |
| T2 | 74 (40.2) | 34 (37.4) |
| T3 | 90 (48.9) | 47 (51.6) |
| T4 | 9 (4.9) | 9 (9.9) |
| Unknown | 6 (3.3) |  |
| **Clinical node status at baseline** |  |  |
| N0 | 60 (32.6) | 29 (31.9) |
| N1 | 90 (48.9) | 45(49.5) |
| N2 | 17 (9.2) | 9 (9.9) |
| N3 | 7 (3.8) | 5 (5.5) |
| Unkown | 10 (5.4) | 3 (3.3) |
| **Histologic Grade** |  |  |
| 1 & 2 | 32 (17.4) | 23 (25.3) |
| 3 | 77 (41.8) | 53 (58.2) |
| Unknown | 75 (40.8) | 15 (16.5) |
| **ER status** |  |  |
| Positive^1^ | 75 (40.8) | 38 (41.8) |
| Negative | 108 (58.7) | 52 (57.1) |
| Unknown | 1 (0.5) | 1 (1.1) |
| **HER-2 status** |  |  |
| Positive ^2^ | 51 (27.4) | 34 (37.4) |
| Negative | 89 (48.4) | 54 (59.3) |
| Unknown | 44 (23.9) | 3 (3.3) |

^1^Cases where >10% of tumor cells stained positive for ER with immunohistochemistry were considered positive. ^2^Cases that showed either 3+ IHC staining or had gene copy number >2.0 were considered HER2-positive.

**Supplementary Table 3**. Two hundred and ninety one affymetrix probes used in TFEC MGP. The gene symbols and descriptions of these probes were obtained by using broad institute’s molecular signatures database v3.0 (http://www.broadinstitute.org/gsea/msigdb/index.jsp) .

| Affymetrix id | gene symbol | description |
| --- | --- | --- |
| 117_at | no mapping |  |
| 200044_at | SFRS9 | splicing factor, arginine/serine-rich 9 |
| 200049_at | MYST2 | MYST histone acetyltransferase 2 |
| 200054_at | ZNF259 | zinc finger protein 259 |
| 200074_s_at | no mapping |  |
| 200087_s_at | TMED2 | transmembrane emp24 domain trafficking protein 2 |
| 200614_at | CLTC | clathrin, heavy chain (Hc) |
| 200617_at | KIAA0152 | KIAA0152 |
| 200803_s_at | TEGT | testis enhanced gene transcript (BAX inhibitor 1) |
| 200804_at | TEGT | testis enhanced gene transcript (BAX inhibitor 1) |
| 200806_s_at | HSPD1 | heat shock 60kDa protein 1 (chaperonin) |
| 200864_s_at | RAB11A | RAB11A, member RAS oncogene family |
| 200869_at | no mapping |  |
| 200925_at | COX6A1 | cytochrome c oxidase subunit VIa polypeptide 1 |
| 200927_s_at | RAB14 | RAB14, member RAS oncogene family |
| 200934_at | DEK | DEK oncogene (DNA binding) |
| 200956_s_at | SSRP1 | structure specific recognition protein 1 |
| 200987_x_at | PSME3 | proteasome (prosome, macropain) activator subunit 3 (PA28 gamma; Ki) |
| 201068_s_at | PSMC2 | proteasome (prosome, macropain) 26S subunit, ATPase, 2 |
| 201138_s_at | SSB | Sjogren syndrome antigen B (autoantigen La) |
| 201144_s_at | EIF2S1 | eukaryotic translation initiation factor 2, subunit 1 alpha, 35kDa |
| 201176_s_at | ARCN1 | archain 1 |
| 201231_s_at | ENO1 | enolase 1, (alpha) |
| 201276_at | RAB5B | RAB5B, member RAS oncogene family |
| 201291_s_at | TOP2A | topoisomerase (DNA) II alpha 170kDa |
| 201323_at | EBNA1BP2 | EBNA1 binding protein 2 |
| 201336_at | VAMP3 | vesicle-associated membrane protein 3 (cellubrevin) |
| 201339_s_at | SCP2 | sterol carrier protein 2 |
| 201370_s_at | CUL3 | cullin 3 |
| 201371_s_at | CUL3 | cullin 3 |
| 201443_s_at | ATP6AP2 | ATPase, H+ transporting, lysosomal accessory protein 2 |
| 201503_at | G3BP | - |
| 201646_at | SCARB2 | scavenger receptor class B, member 2 |
| 201647_s_at | SCARB2 | scavenger receptor class B, member 2 |
| 201662_s_at | ACSL3 | acyl-CoA synthetase long-chain family member 3 |
| 201698_s_at | SFRS9 | splicing factor, arginine/serine-rich 9 |
| 201706_s_at | PEX19 | peroxisomal biogenesis factor 19 |
| 201797_s_at | VARS | valyl-tRNA synthetase |
| 201838_s_at | SUPT7L | suppressor of Ty 7 (S. cerevisiae)-like |
| 202026_at | SDHD | succinate dehydrogenase complex, subunit D, integral membrane protein |
| 202038_at | UBE4A | ubiquitination factor E4A (UFD2 homolog, yeast) |
| 202042_at | HARS | histidyl-tRNA synthetase |
| 202106_at | GOLGA3 | golgi autoantigen, golgin subfamily a, 3 |
| 202136_at | ZMYND11 | zinc finger, MYND domain containing 11 |
| 202137_s_at | ZMYND11 | zinc finger, MYND domain containing 11 |
| 202170_s_at | AASDHPPT | aminoadipate-semialdehyde dehydrogenase-phosphopantetheinyl transferase |
| 202197_at | MTMR3 | myotubularin related protein 3 |
| 202200_s_at | SRPK1 | SFRS protein kinase 1 |
| 202249_s_at | WDR42A | WD repeat domain 42A |
| 202309_at | MTHFD1 | methylenetetrahydrofolate dehydrogenase (NADP+ dependent) 1, methenyltetrahydrofolate cyclohydrolase, formyltetrahydrofolate synthetase |
| 202346_at | HIP2 | huntingtin interacting protein 2 |
| 202384_s_at | TCOF1 | Treacher Collins-Franceschetti syndrome 1 |
| 202385_s_at | TCOF1 | Treacher Collins-Franceschetti syndrome 1 |
| 202433_at | SLC35B1 | solute carrier family 35, member B1 |
| 202448_s_at | ZYG11BL | zyg-11 homolog B (C. elegans)-like |
| 202521_at | CTCF | CCCTC-binding factor (zinc finger protein) |
| 202690_s_at | SNRPD1 | small nuclear ribonucleoprotein D1 polypeptide 16kDa |
| 202696_at | OXSR1 | oxidative-stress responsive 1 |
| 202715_at | CAD | carbamoyl-phosphate synthetase 2, aspartate transcarbamylase, and dihydroorotase |
| 202882_x_at | NOL7 | nucleolar protein 7, 27kDa |
| 202900_s_at | NUP88 | nucleoporin 88kDa |
| 202955_s_at | ARFGEF1 | ADP-ribosylation factor guanine nucleotide-exchange factor 1(brefeldin A-inhibited) |
| 203023_at | HSPC111 | - |
| 203040_s_at | HMBS | hydroxymethylbilane synthase |
| 203095_at | MTIF2 | mitochondrial translational initiation factor 2 |
| 203341_at | CEBPZ | CCAAT/enhancer binding protein zeta |
| 203383_s_at | GOLGA1 | golgi autoantigen, golgin subfamily a, 1 |
| 203384_s_at | GOLGA1 | golgi autoantigen, golgin subfamily a, 1 |
| 203388_at | ARRB2 | arrestin, beta 2 |
| 203405_at | DSCR2 | Down syndrome critical region gene 2 |
| 203462_x_at | EIF3S9 | eukaryotic translation initiation factor 3, subunit 9 eta, 116kDa |
| 203492_x_at | CEP57 | centrosomal protein 57kDa |
| 203529_at | PPP6C | protein phosphatase 6, catalytic subunit |
| 203622_s_at | LOC56902 | - |
| 203694_s_at | DHX16 | DEAH (Asp-Glu-Ala-His) box polypeptide 16 |
| 203707_at | ZNF263 | zinc finger protein 263 |
| 203764_at | DLG7 | discs, large homolog 7 (Drosophila) |
| 203825_at | BRD3 | bromodomain containing 3 |
| 203856_at | VRK1 | vaccinia related kinase 1 |
| 203870_at | USP46 | ubiquitin specific peptidase 46 |
| 203901_at | MAP3K7IP1 | mitogen-activated protein kinase kinase kinase 7 interacting protein 1 |
| 203944_x_at | BTN2A1 | butyrophilin, subfamily 2, member A1 |
| 204028_s_at | RABGAP1 | RAB GTPase activating protein 1 |
| 204175_at | ZNF593 | zinc finger protein 593 |
| 204228_at | PPIH | peptidylprolyl isomerase H (cyclophilin H) |
| 204251_s_at | CEP164 | centrosomal protein 164kDa |
| 204327_s_at | ZNF202 | zinc finger protein 202 |
| 204384_at | GOLGA2 | golgi autoantigen, golgin subfamily a, 2 |
| 204405_x_at | DIMT1L | DIM1 dimethyladenosine transferase 1-like (S. cerevisiae) |
| 204458_at | LYPLA3 | lysophospholipase 3 (lysosomal phospholipase A2) |
| 204640_s_at | SPOP | speckle-type POZ protein |
| 204690_at | STX8 | syntaxin 8 |
| 204905_s_at | EEF1E1 | eukaryotic translation elongation factor 1 epsilon 1 |
| 204977_at | DDX10 | DEAD (Asp-Glu-Ala-Asp) box polypeptide 10 |
| 205176_s_at | ITGB3BP | integrin beta 3 binding protein (beta3-endonexin) |
| 205252_at | ZNF174 | zinc finger protein 174 |
| 205324_s_at | FTSJ1 | FtsJ homolog 1 (E. coli) |
| 205395_s_at | MRE11A | MRE11 meiotic recombination 11 homolog A (S. cerevisiae) |
| 205423_at | AP1B1 | adaptor-related protein complex 1, beta 1 subunit |
| 205545_x_at | DNAJC8 | DnaJ (Hsp40) homolog, subfamily C, member 8 |
| 205677_s_at | DLEU1 | deleted in lymphocytic leukemia, 1 |
| 205996_s_at | AK2 | adenylate kinase 2 |
| 206098_at | ZBTB6 | zinc finger and BTB domain containing 6 |
| 206174_s_at | PPP6C | protein phosphatase 6, catalytic subunit |
| 206499_s_at | RCC1 | regulator of chromosome condensation 1 |
| 206653_at | no mapping |  |
| 206752_s_at | DFFB | DNA fragmentation factor, 40kDa, beta polypeptide (caspase-activated DNase) |
| 206968_s_at | NFRKB | nuclear factor related to kappaB binding protein |
| 207127_s_at | HNRPH3 | heterogeneous nuclear ribonucleoprotein H3 (2H9) |
| 207458_at | C8ORF51 | chromosome 8 open reading frame 51 |
| 207573_x_at | ATP5L | ATP synthase, H+ transporting, mitochondrial F0 complex, subunit G |
| 207668_x_at | PDIA6 | protein disulfide isomerase family A, member 6 |
| 208002_s_at | ACOT7 | acyl-CoA thioesterase 7 |
| 208152_s_at | DDX21 | DEAD (Asp-Glu-Ala-Asp) box polypeptide 21 |
| 208398_s_at | TBPL1 | TBP-like 1 |
| 208627_s_at | YBX1 | Y box binding protein 1 |
| 208688_x_at | EIF3S9 | eukaryotic translation initiation factor 3, subunit 9 eta, 116kDa |
| 208696_at | CCT5 | chaperonin containing TCP1, subunit 5 (epsilon) |
| 208736_at | ARPC3 | actin related protein 2/3 complex, subunit 3, 21kDa |
| 208737_at | ATP6V1G1 | ATPase, H+ transporting, lysosomal 13kDa, V1 subunit G1 |
| 208746_x_at | ATP5L | ATP synthase, H+ transporting, mitochondrial F0 complex, subunit G |
| 208756_at | EIF3S2 | eukaryotic translation initiation factor 3, subunit 2 beta, 36kDa |
| 208841_s_at | G3BP2 | - |
| 208897_s_at | DDX18 | DEAD (Asp-Glu-Ala-Asp) box polypeptide 18 |
| 208910_s_at | C1QBP | complement component 1, q subcomponent binding protein |
| 208927_at | SPOP | speckle-type POZ protein |
| 208959_s_at | TXNDC4 | thioredoxin domain containing 4 (endoplasmic reticulum) |
| 209104_s_at | NOLA2 | nucleolar protein family A, member 2 (H/ACA small nucleolar RNPs) |
| 209196_at | WDR46 | WD repeat domain 46 |
| 209221_s_at | OSBPL2 | oxysterol binding protein-like 2 |
| 209333_at | ULK1 | unc-51-like kinase 1 (C. elegans) |
| 209390_at | TSC1 | tuberous sclerosis 1 |
| 209421_at | MSH2 | mutS homolog 2, colon cancer, nonpolyposis type 1 (E. coli) |
| 209630_s_at | FBXW2 | F-box and WD-40 domain protein 2 |
| 209654_at | KIAA0947 | - |
| 209669_s_at | SERBP1 | SERPINE1 mRNA binding protein 1 |
| 209798_at | NPAT | nuclear protein, ataxia-telangiectasia locus |
| 209820_s_at | TBL3 | transducin (beta)-like 3 |
| 209862_s_at | CEP57 | centrosomal protein 57kDa |
| 210005_at | GART | phosphoribosylglycinamide formyltransferase, phosphoribosylglycinamide synthetase, phosphoribosylaminoimidazole synthetase |
| 210075_at | 2-Mar | membrane-associated ring finger (C3HC4) 2 |
| 210097_s_at | NOL7 | nucleolar protein 7, 27kDa |
| 210098_s_at | no mapping |  |
| 210110_x_at | HNRPH3 | heterogeneous nuclear ribonucleoprotein H3 (2H9) |
| 210175_at | C2ORF3 | chromosome 2 open reading frame 3 |
| 210453_x_at | ATP5L | ATP synthase, H+ transporting, mitochondrial F0 complex, subunit G |
| 210466_s_at | SERBP1 | SERPINE1 mRNA binding protein 1 |
| 210581_x_at | PATZ1 | POZ (BTB) and AT hook containing zinc finger 1 |
| 210633_x_at | KRT10 | keratin 10 (epidermolytic hyperkeratosis; keratosis palmaris et plantaris) |
| 211150_s_at | DLAT | dihydrolipoamide S-acetyltransferase (E2 component of pyruvate dehydrogenase complex) |
| 211392_s_at | PATZ1 | POZ (BTB) and AT hook containing zinc finger 1 |
| 211493_x_at | DTNA | dystrobrevin, alpha |
| 211503_s_at | RAB14 | RAB14, member RAS oncogene family |
| 211623_s_at | FBL | fibrillarin |
| 211787_s_at | EIF4A1 | eukaryotic translation initiation factor 4A, isoform 1 |
| 211979_at | GPR107 | G protein-coupled receptor 107 |
| 212053_at | KIAA0251 | - |
| 212068_s_at | KIAA0515 | KIAA0515 |
| 212295_s_at | SLC7A1 | solute carrier family 7 (cationic amino acid transporter, y+ system), member 1 |
| 212319_at | RUTBC1 | RUN and TBC1 domain containing 1 |
| 212348_s_at | AOF2 | amine oxidase (flavin containing) domain 2 |
| 212367_at | FEM1B | fem-1 homolog b (C. elegans) |
| 212378_at | GART | phosphoribosylglycinamide formyltransferase, phosphoribosylglycinamide synthetase, phosphoribosylaminoimidazole synthetase |
| 212400_at | FAM102A | family with sequence similarity 102, member A |
| 212403_at | UBE3B | ubiquitin protein ligase E3B |
| 212404_s_at | UBE3B | ubiquitin protein ligase E3B |
| 212518_at | PIP5K1C | phosphatidylinositol-4-phosphate 5-kinase, type I, gamma |
| 212547_at | FLJ35348 | - |
| 212568_s_at | DLAT | dihydrolipoamide S-acetyltransferase (E2 component of pyruvate dehydrogenase complex) |
| 212603_at | MRPS31 | mitochondrial ribosomal protein S31 |
| 212604_at | MRPS31 | mitochondrial ribosomal protein S31 |
| 212653_s_at | EHBP1 | EH domain binding protein 1 |
| 212725_s_at | no mapping |  |
| 212846_at | KIAA0179 | KIAA0179 |
| 212858_at | PAQR4 | progestin and adipoQ receptor family member IV |
| 212920_at | no mapping |  |
| 213028_at | NFRKB | nuclear factor related to kappaB binding protein |
| 213097_s_at | ZRF1 | zuotin related factor 1 |
| 213141_at | PSKH1 | protein serine kinase H1 |
| 213149_at | DLAT | dihydrolipoamide S-acetyltransferase (E2 component of pyruvate dehydrogenase complex) |
| 213185_at | KIAA0556 | KIAA0556 |
| 213196_at | ZNF629 | zinc finger protein 629 |
| 213302_at | PFAS | phosphoribosylformylglycinamidine synthase (FGAR amidotransferase) |
| 213473_at | BRAP | BRCA1 associated protein |
| 213588_x_at | RPL14 | ribosomal protein L14 |
| 213743_at | CCNT2 | cyclin T2 |
| 213864_s_at | NAP1L1 | nucleosome assembly protein 1-like 1 |
| 214011_s_at | HSPC111 | - |
| 214070_s_at | ATP10B | ATPase, Class V, type 10B |
| 214138_at | ZNF79 | zinc finger protein 79 |
| 214209_s_at | ABCB9 | ATP-binding cassette, sub-family B (MDR/TAP), member 9 |
| 214317_x_at | RPS9 | ribosomal protein S9 |
| 214448_x_at | NFKBIB | nuclear factor of kappa light polypeptide gene enhancer in B-cells inhibitor, beta |
| 215113_s_at | SENP3 | SUMO1/sentrin/SMT3 specific peptidase 3 |
| 215136_s_at | EXOSC8 | exosome component 8 |
| 215207_x_at | no mapping |  |
| 215696_s_at | KIAA0310 | KIAA0310 |
| 215728_s_at | ACOT7 | acyl-CoA thioesterase 7 |
| 215766_at | GSTA1 | glutathione S-transferase A1 |
| 215982_s_at | DOM3Z | dom-3 homolog Z (C. elegans) |
| 216226_at | TAF4B | TAF4b RNA polymerase II, TATA box binding protein (TBP)-associated factor, 105kDa |
| 216294_s_at | KIAA1109 | KIAA1109 |
| 216326_s_at | HDAC3 | histone deacetylase 3 |
| 216389_s_at | WDR23 | WD repeat domain 23 |
| 216961_s_at | RPAIN | RPA interacting protein |
| 217106_x_at | DIMT1L | DIM1 dimethyladenosine transferase 1-like (S. cerevisiae) |
| 217294_s_at | ENO1 | enolase 1, (alpha) |
| 217445_s_at | GART | phosphoribosylglycinamide formyltransferase, phosphoribosylglycinamide synthetase, phosphoribosylaminoimidazole synthetase |
| 217747_s_at | RPS9 | ribosomal protein S9 |
| 217777_s_at | PTPLAD1 | protein tyrosine phosphatase-like A domain containing 1 |
| 217939_s_at | no mapping |  |
| 217994_x_at | CPSF3L | cleavage and polyadenylation specific factor 3-like |
| 218104_at | TEX10 | testis expressed sequence 10 |
| 218107_at | WDR26 | WD repeat domain 26 |
| 218155_x_at | TSR1 | TSR1, 20S rRNA accumulation, homolog (S. cerevisiae) |
| 218156_s_at | TSR1 | TSR1, 20S rRNA accumulation, homolog (S. cerevisiae) |
| 218190_s_at | UCRC | - |
| 218244_at | NOL8 | nucleolar protein 8 |
| 218278_at | WDR74 | WD repeat domain 74 |
| 218333_at | DERL2 | Der1-like domain family, member 2 |
| 218350_s_at | GMNN | geminin, DNA replication inhibitor |
| 218512_at | WDR12 | WD repeat domain 12 |
| 218525_s_at | HIF1AN | hypoxia-inducible factor 1, alpha subunit inhibitor |
| 218527_at | APTX | aprataxin |
| 218566_s_at | CHORDC1 | cysteine and histidine-rich domain (CHORD)-containing 1 |
| 218580_x_at | no mapping |  |
| 218597_s_at | ZCD1 | zinc finger, CDGSH-type domain 1 |
| 218626_at | EIF4ENIF1 | eukaryotic translation initiation factor 4E nuclear import factor 1 |
| 218710_at | TTC27 | tetratricopeptide repeat domain 27 |
| 218754_at | NOL9 | nucleolar protein 9 |
| 218774_at | DCPS | decapping enzyme, scavenger |
| 218830_at | RPL26L1 | ribosomal protein L26-like 1 |
| 218877_s_at | C6ORF75 | chromosome 6 open reading frame 75 |
| 218886_at | PAK1IP1 | PAK1 interacting protein 1 |
| 218982_s_at | MRPS17 | mitochondrial ribosomal protein S17 |
| 219081_at | ANKHD1 | ankyrin repeat and KH domain containing 1 |
| 219086_at | C14ORF131 | chromosome 14 open reading frame 131 |
| 219098_at | MYBBP1A | MYB binding protein (P160) 1a |
| 219122_s_at | THG1L | tRNA-histidine guanylyltransferase 1-like (S. cerevisiae) |
| 219220_x_at | MRPS22 | mitochondrial ribosomal protein S22 |
| 219293_s_at | GTPBP9 | GTP-binding protein 9 (putative) |
| 219336_s_at | ASCC1 | activating signal cointegrator 1 complex subunit 1 |
| 219374_s_at | ALG9 | asparagine-linked glycosylation 9 homolog (S. cerevisiae, alpha- 1,2-mannosyltransferase) |
| 219679_s_at | WAC | WW domain containing adaptor with coiled-coil |
| 219784_at | FBXO31 | F-box protein 31 |
| 220223_at | C17ORF41 | chromosome 17 open reading frame 41 |
| 220255_at | FANCE | Fanconi anemia, complementation group E |
| 220419_s_at | USP25 | ubiquitin specific peptidase 25 |
| 220606_s_at | C17ORF48 | chromosome 17 open reading frame 48 |
| 220943_s_at | PRO1853 | - |
| 220964_s_at | RAB1B | RAB1B, member RAS oncogene family |
| 221096_s_at | TMCO6 | transmembrane and coiled-coil domains 6 |
| 221158_at | C21ORF66 | chromosome 21 open reading frame 66 |
| 221230_s_at | ARID4B | AT rich interactive domain 4B (RBP1- like) |
| 221255_s_at | TMEM93 | transmembrane protein 93 |
| 221263_s_at | SF3B5 | splicing factor 3b, subunit 5, 10kDa |
| 221434_s_at | C14ORF156 | chromosome 14 open reading frame 156 |
| 221488_s_at | CUTA | cutA divalent cation tolerance homolog (E. coli) |
| 221504_s_at | ATP6V1H | ATPase, H+ transporting, lysosomal 50/57kDa, V1 subunit H |
| 221517_s_at | CRSP6 | cofactor required for Sp1 transcriptional activation, subunit 6, 77kDa |
| 221580_s_at | JOSD3 | Josephin domain containing 3 |
| 221691_x_at | NPM1 | nucleophosmin (nucleolar phosphoprotein B23, numatrin) |
| 221699_s_at | DDX50 | DEAD (Asp-Glu-Ala-Asp) box polypeptide 50 |
| 221700_s_at | UBA52 | ubiquitin A-52 residue ribosomal protein fusion product 1 |
| 221712_s_at | WDR74 | WD repeat domain 74 |
| 221836_s_at | NIBP | - |
| 221923_s_at | NPM1 | nucleophosmin (nucleolar phosphoprotein B23, numatrin) |
| 221987_s_at | TSR1 | TSR1, 20S rRNA accumulation, homolog (S. cerevisiae) |
| 222000_at | C1ORF174 | chromosome 1 open reading frame 174 |
| 222029_x_at | PFDN6 | prefoldin subunit 6 |
| 222163_s_at | SPATA5L1 | spermatogenesis associated 5-like 1 |
| 222200_s_at | BSDC1 | BSD domain containing 1 |
| 222229_x_at | no mapping |  |
| 222244_s_at | TUG1 | - |
| 33760_at | PEX14 | peroxisomal biogenesis factor 14 |
| 35436_at | GOLGA2 | golgi autoantigen, golgin subfamily a, 2 |
| 37079_at | YDD19 | - |
| 37831_at | SIPA1L3 | signal-induced proliferation-associated 1 like 3 |
| 38157_at | DOM3Z | dom-3 homolog Z (C. elegans) |
| 40829_at | WDTC1 | WD and tetratricopeptide repeats 1 |
| 41512_at | no mapping |  |
| 43977_at | TMEM161A | transmembrane protein 161A |
| 44563_at | WDR79 | WD repeat domain 79 |
| 45526_g_at | FLJ14154 | - |
| 46256_at | SPSB3 | splA/ryanodine receptor domain and SOCS box containing 3 |
| 46270_at | UBAP1 | ubiquitin associated protein 1 |
| 50376_at | ZNF444 | zinc finger protein 444 |
| 56829_at | NIBP | - |
| 61874_at | C9ORF7 | chromosome 9 open reading frame 7 |
| 64440_at | IL17RC | interleukin 17 receptor C |
| 77508_r_at | no mapping |  |

**Supplementary Table 4**. Identified enriched pathways.

| Gene sets | q-value |
| --- | --- |
| BERENJENO_TRANSFORMED_BY_RHOA_UP | 0.000 |
| LASTOWSKA_NEUROBLASTOMA_COPY_NUMBER_DN | 0.000 |
| LINDGREN_BLADDER_CANCER_WITH_LOH_IN_CHR9Q | 0.000 |
| SCHLOSSER_MYC_TARGETS_REPRESSED_BY_SERUM | 0.000 |
| MANALO_HYPOXIA_DN | 0.000 |
| SCHUHMACHER_MYC_TARGETS_UP | 0.000 |
| MOREAUX_MULTIPLE_MYELOMA_BY_TACI_DN | 0.000 |
| DANG_MYC_TARGETS_UP | 0.000 |
| WONG_EMBRYONIC_STEM_CELL_CORE | 0.000 |
| REACTOME_GTP_HYDROLYSIS_AND_JOINING_OF_THE_60S_RIBOSOMAL_SUBUNIT | 0.000 |
| BENPORATH_ES_1 | 0.001 |
| REACTOME_TRANSLATION | 0.001 |
| REACTOME_FORMATION_OF_A_POOL_OF_FREE_40S_SUBUNITS | 0.002 |
| REACTOME_GENE_EXPRESSION | 0.002 |
| REACTOME_METABOLISM_OF_PROTEINS | 0.003 |
| MILI_PSEUDOPODIA_HAPTOTAXIS_UP | 0.004 |
| MUELLER_PLURINET | 0.010 |
| YAO_TEMPORAL_RESPONSE_TO_PROGESTERONE_CLUSTER_14 | 0.010 |
| RHEIN_ALL_GLUCOCORTICOID_THERAPY_DN | 0.011 |
| KIM_MYC_AMPLIFICATION_TARGETS_UP | 0.011 |
| REACTOME_PEPTIDE_CHAIN_ELONGATION | 0.021 |
| LIU_SOX4_TARGETS_DN | 0.022 |
| REACTOME_VIRAL_MRNA_TRANSLATION | 0.022 |
| BILD_MYC_ONCOGENIC_SIGNATURE | 0.023 |
| KEGG_RIBOSOME | 0.024 |
| MOREAUX_B_LYMPHOCYTE_MATURATION_BY_TACI_DN | 0.031 |
| YAO_TEMPORAL_RESPONSE_TO_PROGESTERONE_CLUSTER_11 | 0.032 |
| REACTOME_TRANSLATION_INITIATION_COMPLEX_FORMATION | 0.032 |
| REACTOME_REGULATION_OF_GENE_EXPRESSION_IN_BETA_CELLS | 0.040 |
| ENK_UV_RESPONSE_KERATINOCYTE_DN | 0.050 |
| SCHLOSSER_MYC_TARGETS_AND_SERUM_RESPONSE_DN | 0.051 |
| PRAMOONJAGO_SOX4_TARGETS_DN | 0.054 |
| CHNG_MULTIPLE_MYELOMA_HYPERPLOID_UP | 0.054 |
| REACTOME_REGULATION_OF_BETA_CELL_DEVELOPMENT | 0.054 |
| WINNEPENNINCKX_MELANOMA_METASTASIS_UP | 0.056 |
| LIU_NASOPHARYNGEAL_CARCINOMA | 0.058 |
| PUJANA_BREAST_CANCER_WITH_BRCA1_MUTATED_UP | 0.058 |
| BIDUS_METASTASIS_UP | 0.070 |
| GRADE_COLON_CANCER_UP | 0.070 |
| REACTOME_INFLUENZA_LIFE_CYCLE | 0.072 |
| BLALOCK_ALZHEIMERS_DISEASE_INCIPIENT_DN | 0.084 |
| ZHANG_BREAST_CANCER_PROGENITORS_UP | 0.086 |
| REACTOME_FORMATION_OF_THE_TERNARY_COMPLEX_AND_SUBSEQUENTLY_THE_43S_COMPLEX | 0.086 |
| REACTOME_INFLUENZA_VIRAL_RNA_TRANSCRIPTION_AND_REPLICATION | 0.086 |
